# Impact of Diabetic Ketoacidosis on Thyroid Function in Patients with Diabetes Mellitus

**DOI:** 10.1155/2021/2421091

**Published:** 2021-03-20

**Authors:** Yuling Xing, Jinhu Chen, Guangyao Song, Liying Zhao, Huijuan Ma

**Affiliations:** ^1^Department of Endocrinology, Hebei General Hospital, Shijiazhuang 050017, China; ^2^Graduate School of Hebei Medical University, Shijiazhuang 050017, China; ^3^Hebei Key Laboratory of Metabolic Diseases, Hebei General Hospital, Shijiazhuang, Hebei 050051, China; ^4^Department of Internal Medicine, Hebei Medical University, Shijiazhuang, Hebei 050017, China

## Abstract

**Background:**

Changes in thyroid function in diabetes patients who developed diabetic ketoacidosis (DKA) still need to be fully elucidated. The aim of this study was to systematically review available data on the relationship between thyroid function and DKA in diabetes patients who developed DKA.

**Methods:**

Electronic databases (PubMed, EMBASE, Cochrane Library, and China Academic Journal Full-text Database (CNKI)) were searched systematically to search relevant literature before December 2020. The mean ± standard deviation and 95% confidence interval (95% CI) were used for evaluation, and sensitivity analysis was performed. Publication bias was estimated by funnel plot, Egger's test, and Begger's test.

**Results:**

29 studies were included in the meta-analysis, and the indicators (T4, T3, FT3, FT4, TSH, T3RU, and rT3) of patients with DKA were compared and analyzed. The results of this study showed that the levels of T4, T3, FT3, FT4, and TSH were decreased and the level of rT3 was increased in patients with DKA. Compared with after treatment, the levels of T4, T3, FT3, and FT4 in patients with DKA were decreased before treatment, while the levels of rT3 were increased, and there was no significant difference in changes of TSH. With the aggravation of DKA, the levels of T4, T3, FT3, and FT4 will further decrease, while the changes of TSH have no statistical difference.

**Conclusion:**

Thyroid function changed in diabetic patients with DKA. It changed with the severity of DKA. This condition may be transient, preceding further recovery of DKA.

## 1. Introduction

Diabetic ketoacidosis (DKA) is an acute life-threatening complication of diabetes. It is not only a sign of acute absolute insulin deficiency in type 1 diabetes mellitus (T1DM) but also increasingly seen in patients with type 2 diabetes mellitus. In patients with diabetes, ketoacidosis is caused by an acute decrease in insulin secretion and action in a severe insulin resistant state [1]. From 2002 to 2010 in the United States, about 30% of adolescents newly diagnosed with T1DM developed DKA [2]. The prevalence of DKA estimated at the onset of type 2 diabetes is quite different. African-American youth in Cincinnati and Arkansas was 41.4% [3] and 16% [4]. Statistics showed that thyroid dysfunction in people with diabetes is 2-3 times higher than people without diabetes [5]. The effect of nonthyroid diseases on thyroid function has been studied in anorexia nervosa, liver disease, kidney disease, and many other diseases [6]. Since the 1970s, it has been reported that acute disease can cause a variety of changes in the levels of thyroid hormones in patients who were not previously diagnosed with intrinsic thyroid disease. These changes are nonspecific and are related to the severity of the disease [7]. Diabetes can have a definite effect on thyroid function in various ways, leading to changes in the levels of thyroid hormones, including immunological mechanisms, cytokine pathways, and regulatory pathways of the hypothalamic-pituitary-thyroid axis [8]. When DKA occurred in patients with diabetes, the changes in thyroid function has received a great deal of attention from researchers. At present, there are limited studies on the changes in levels of thyroid hormone in patients with DKA. DKA and its implication in the thyroid function has not been adequately reviewed. The study aimed to analyze the changes in the levels of thyroid hormones in patients with DKA and the relationship between the changes and the severity of DKA.

## 2. Materials and Methods

### 2.1. Literature Search Strategy

Diabetic ketoacidosis, related indicators reflecting thyroid function (free triiodothyronine (FT3), free thyroxine (FT4), triiodothyronine (T3), thyroxine (T4), thyroid-stimulating hormone (TSH), T3 resin uptake (T3RU), and reverse triiodothyronine (rT3)) as subject terms and keywords for joint search. All relevant literature published before December 2020 was searched in PubMed, EMBASE, Cochrane Library, and CNKI.

### 2.2. Inclusion Criteria

(1) The article related to patients with DKA; (2) involving the changes of thyroid function indicators in patients with DKA before and after treatment or between the diabetic patients with and without DKA and providing the exact sample size and data on various indicators of thyroid function; and (3) the diagnosis of diabetic ketoacidosis is clear [9].

### 2.3. Exclusion Criteria

(1) The data of literature are incomplete and the information is not enough to calculate the statistics of this study; (2) case reports; (3) repeated articles; and (4) studies limited to animals.

### 2.4. Literature Screening

Two researchers independently screened the literature, extracted data, and cross-checked. If there is a disagreement on the results, they would discuss it together or resolve it by a third senior researcher. In the study, data were extracted from the literature finally included in the meta-analysis using a premade data extraction table. The extracted content included the first author, year of publication, study area, sample size, mean ± standard deviation of thyroid function indicators, inclusion criteria, exclusion criteria, DKA diagnostic cutoff point, the determination method of thyroid hormone, therapeutic approach, and duration of treatment of DKA (Table 1).

### 2.5. Statistical Analysis

According to the requirements of meta-analysis, the data were sorted out, the database was established, the data were carefully checked, and the standardized mean difference (SMD) and 95% CI were used to quantitatively analyze the measurement data. I2 was used to quantitatively test the heterogeneity among the studies. If I2 ≤ 50%, it was considered that the heterogeneity was not statistically significant, and the fixed effect model was used to analyze; on the contrary, if I2 > 50%, the heterogeneity was considered to be statistically significant, and the random effect model was used to analyze. Sensitivity analysis was performed to ensure the stability of the meta-analysis results. Funnel plot and Egger's test were used to evaluate publication bias, and *p* < 0.05 was considered as statistically significant, indicating that publication bias was not excluded. The trim-and-fill method was used to estimate the effect of publication bias on the interpretation of the results.

## 3. Result

### 3.1. Literature Search Results

**Figure 1:**
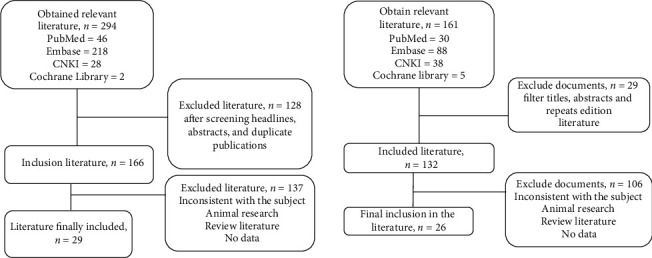
The process of study selection.

314 related studies were initially retrieved based on keywords and subject terms, and finally, 29 studies met the predetermined inclusion and exclusion criteria (Figure 1). 17 studies evaluated the changes of thyroid function before and after treatment in patients with DKA, 17 studies evaluated the difference of thyroid function between patients with diabetes with and without DKA, and 3 studies related to the changes of thyroid function with different severities of DKA. The relevant literature was published from 1978 to 2018 (Tables 1–3).

### 3.2. Meta-Analysis Results

#### 3.2.1. Comparison of Thyroid Function between Patients with Diabetes with and without DKA

**Figure 2:**
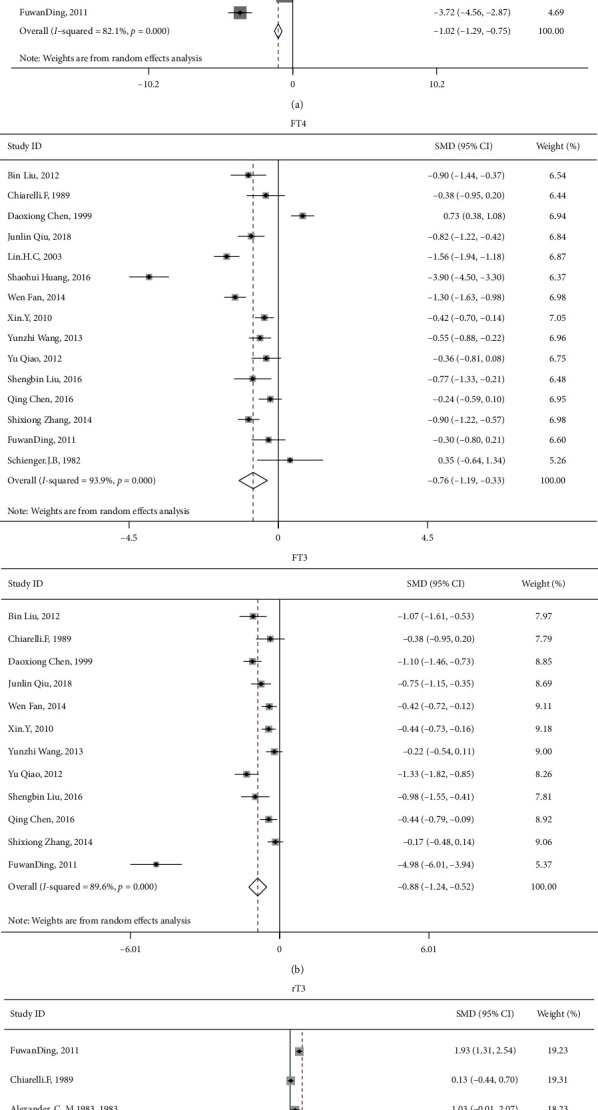
Forest plot of T4, T3, FT4, FT3, rT3, and TSH compared with patients with DKA and diabetes.

**Figure 3:**
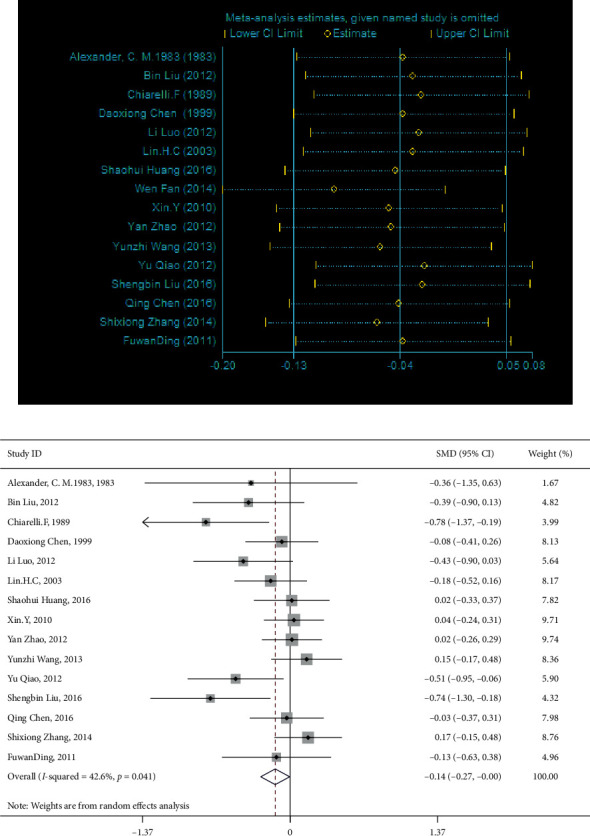
Sensitivity analysis of TSH.

**Figure 4:**
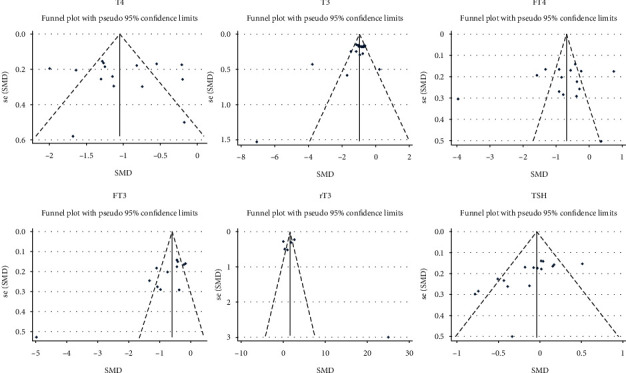
Funnel plot of T4, T3, FT4, FT3, rT3, and TSH compared with patients with DKA and diabetes.

15 studies involved the comparison of T4 between patients with diabetes with and without DKA, involving 751 patients with DKA and 817 patients with diabetes but without DKA; 16 studies involved the comparison of T3, involving 755 patients with DKA and 828 patients with diabetes but without DKA; 15 studies involved the comparison of FT4, involving 790 patients with DKA and 876 patients with diabetes but without DKA; 12 studies involved the comparison of FT3, involving 643 patients with DKA and 744 patients with diabetes but without DKA; 16 studies involved the comparison of TSH, involving 848 patients with diabetes and DKA and 981 patients with diabetes but without DKA; and 6 studies involved the comparison of rT3, involving 135 patients with DKA and 194 patients with diabetes but without DKA. The results showed that compared with patients with diabetes, patients with DKA had lower levels of T4, T3, FT4, and FT3 and higher level of rT3. The difference was statistically significant (T4 : I2 = 83.9%, *p* < 0.01, *Z* = 7.2, *p* < 0.01, SMD = −1.030, 95% CI: −1.310 to −0.749; T3 : I2 = 82.1%, *p* < 0.01, *Z* = 7.4, *p* < 0.01, SMD = −1.022, 95% CI: −1.292 to −0.751; FT4 : I2 = 93.9%, *p* < 0.01, *Z* = 3.45, *p* < 0.01, SMD = −0.758, 95% CI: −1.189 to −0.327; FT3 : I2 = 89.6%, *p* < 0.01, *Z* = 4.82, *p* < 0.01, SMD = −0.884, 95% CI: −1.243 to −0.524; rT3 : I2 = 95.8%, *p* < 0.01, *Z* = 3.15, *p* < 0.01, SMD = 2.534, 95% CI: 0.956 to 4.112; TSH : I2 = 61.1%, *p* < 0.01, *Z* = 1.33, *p*=0.185, SMD = −0.106, 95% CI: −0.261 to 0.05; Figure 2). There was no statistical difference in TSH between patients with diabetes with and without DKA. After sensitivity analysis, the result showed that TSH was significantly different (I2 = 42.6%, *p* < 0.05, *Z* = 2.01, *p* < 0.05, SMD = −0.138, 95% CI: −0.273 to −0.003 Figure 3). Therefore, patients with DKA have lower levels of T4, T3, FT4, FT3, and TSH and higher level of rT3. Egger's test (T4, *p*=0.861; FT4, *p*=0.504; rT3, *p*=0.445) showed that there was no obvious publication bias. Further analysis by the cut-and-fill method showed that the publication bias (T3, *p*=0.043; FT3, *p*=0.003; TSH, 0.003) did not affect the estimator. It is more certain that the effect estimates obtained in the meta-analysis are effective. The funnel plot is shown in Figure 4.

#### 3.2.2. Comparison of Thyroid Function before and after Treatment in Patients with Diabetes and DKA

**Figure 5:**
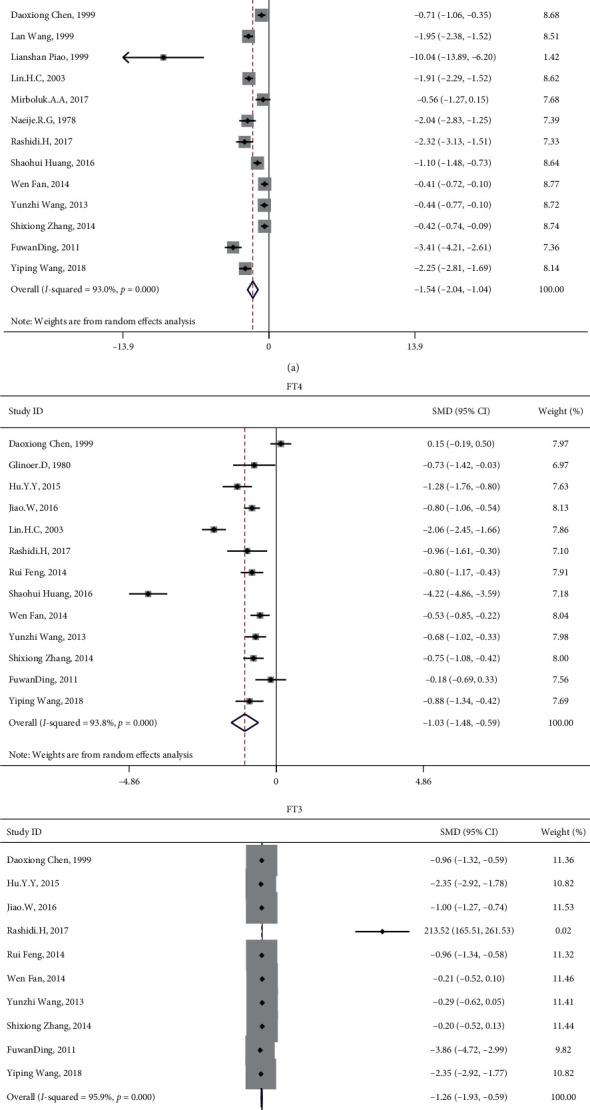
Forest plot of T4, T3, FT4, FT3, and rT3 compared with patients with DKA before and after treatment.

**Figure 6:**
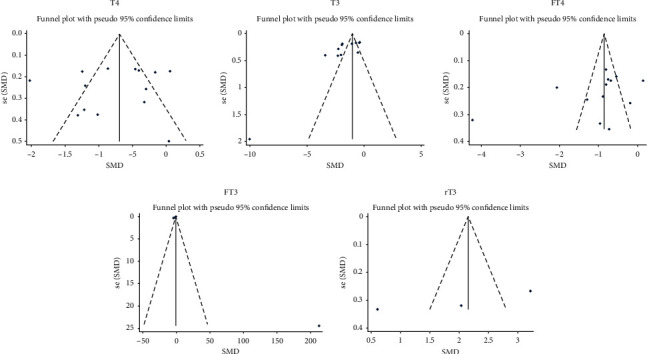
Funnel plot of T4, T3, FT4, FT3, and rT3 compared with patients with DKA before and after treatment.

14 studies involved the comparison of T4 before and after treatment in patients with DKA, including a total of 640 patients with DkA; 13 studies involved the comparison of T3 before and after treatment, including a total of 623 patients with DkA; 13 studies involved the comparison of FT4 before and after treatment, including a total of 755 patients with DkA; 10 studies involved the comparison of FT3 before and after treatment, including a total of 599 patients with DkA; 15 studies involved the comparison of TSH before and after treatment, including a total of 832 patients with DkA; 5 studies involved the comparison of T3RU before and after treatment, including a total of 148 patients with DkA; and 3 studies involved the comparison of rT3 before and after treatment, including a total of 114 patients with DkA. The results showed that patients with DKA had lower levels of T4, T3, FT4, and FT3 and higher level of rT3 compared with after treatment. The difference was statistically significant (T4 : I2 = 86.2%, *p* < 0.01, *Z* = 4.50, *p* < 0.01, SMD = −0.742, 95% CI: −1.066 to 0.419; T3 : I2 = 93%, *p* < 0.01, *Z* = 6.04, *p* < 0.01, SMD = −1.538, 95% CI: −2.037 to −1.039; FT4 : I2 = 93.8%, *p* < 0.01, *Z* = 4.52, *p* < 0.01, SMD = −1.035, 95% CI: −1.483 to −0.586; FT3 : I2 = 95.9%, *p* < 0.01, *Z* = 3.68, *p* < 0.01, SMD = −1.258, 95% CI: −1.926 to −0.589; rT3 : I2 = 94.7%, *p* < 0.01, *Z* = 2.57, *p*=0.01, SMD = 1.967, 95% CI: 0.467 to 3.467 Figure 5). There was no significant difference in TSH and T3RU in patients with DKA before and after treatment. Egger's test (T4, *p*=0.566; T3RU, *p*=0.243; FT4, *p*=0.175; FT3, *p*=0.988; TSH, 0.599; rT3, *p*=0.236) showed that there was no obvious publication bias, further analysis by the trim-and-fill method showed that the publication bias (T3, *p*=0.006) did not affect the estimator, and it was more certain that the effect estimation obtained in the meta-analysis was effective. The funnel plot is shown in Figure 6.

#### 3.2.3. Comparison of Severity of DKA and Thyroid Function in Patients with Diabetes and DKA

**Figure 7:**
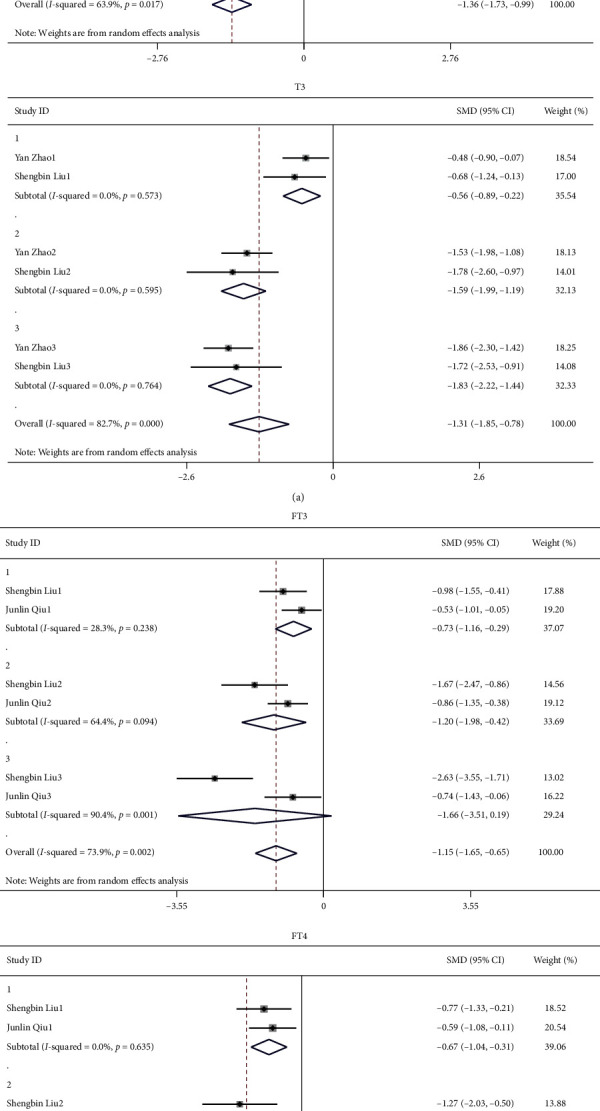
Comparison of severity of DKA and thyroid function in patients with diabetes and DKA.

Three studies involved the comparison of the severity of DKA with thyroid function. The results showed that as the degree of DKA aggravated, the levels of T4, T3, FT4, and FT3 further decreased. The level of TSH increased with the aggravation of DKA, but it was not statistically significant (Figure 7).

## 4. Discussion

This meta-analysis study showed that the levels of T4, T3, FT3, FT4, and TSH were lower and the level of rT3 was higher in patients with DKA compared with patients with diabetes but not DKA. The levels of T4, T3, FT3, and FT4 were lower and the level of rT3 was higher compared with after treatment in patients with diabetes and DKA. As the aggravation of DKA, the levels of T4, T3, FT3, and FT4 would further decrease, but there was no statistical difference in the change of TSH.

DKA can affect the function of the hypothalamus-pituitary-thyroid axis directly or indirectly due to various factors such as relatively insufficient insulin secretion and metabolic disorders, thus affecting thyroid function [38]. Piconi et al. found that large blood glucose fluctuations trigger the production of nitrotyrosine and induce the expression of adhesion molecules and IL-6 [39]. The release of a large number of cytokines acted on the hypothalamus-pituitary-thyroid axis through a variety of ways, which can also affect the synthesis, secretion, metabolism, and feedback of thyroid hormones [40]. An increase in cytokines such as IL-6 synchronizing with a low T3 level is often observed which may cause hypothalamus involvement [41]. The body's caloric intake is seriously insufficient in patients with DKA, leading to hypoxia in the cells, which reduced the biological activity of 5′-deiodinase, resulting in a significant reduction in the conversion of T4 to T3, and a significant reduction in the levels and activity of thyroid hormones [42]. Studies have shown that T1DM and thyroid diseases have a common genetic basis [43]. There is a significant positive correlation between serum TSH and antithyroid antibodies (TRAb, TPOAb, and TGAb) in patients with T2DM, suggesting that abnormal thyroid function in patients with T2DM is autoimmune-mediated pathogenesis [44].

Studies also found that the severity of impaired hypothalamus-hypophysial-thyroid regulation seems to be related to the degree of metabolic disorders regardless of the presence of antithyroid antibodies [45]. Previous studies have shown that the levels of serum T3 and T4 are related to the severity of the disease [46, 47]. Similarly, Balsamo et al. showed that changes in hormone levels are usually related to the severity of metabolic disorders, among which thyroid function is one of the most serious disorders. The hypothalamus-pituitary-thyroid axis showed variable damage, which was defined as nonthyroid disease syndrome (NTIS) [45]. The relationship between the degree of NTIS and the severity of metabolic disorders has previously been reported in adults and children [48–51]. NTIS is now more commonly used to describe a typical change in the serum levels of thyroid-related hormones that may occur after an acute or chronic disease not caused by intrinsic abnormalities in thyroid function. Changes in the hypothalamic-pituitary-thyroid axis also occur in diseases, usually associated with low levels of T3, which gave rise to the term “low T3 syndrome” [52].

It was now well known that most circulating T3 and almost all rT3 came from the peripheral deiodination of T4 [53, 54]. Pittman et al. found that DKA played a certain role in the peripheral transformation of T4 [55]. The moderate decrease in serum T4 observed in patients with DKA has been described previously, which was corrected after treatment, and it seemed to be due to acquired deficiency of T4 binding to serum protein [56]. The factors of dietary, especially carbohydrates, played an important role in the regulation of T3 [57, 58]. The presence of carbohydrate deprivation in DKA seemed to rapidly inhibit the deiodination of T4 by type 1 iodothyronine-deiodinase in the liver, thereby inhibiting the production of T3 and preventing the metabolism of rT3 [59]. Carbohydrate deprivation will lead to a decrease in basal metabolic rate. The decrease in thyroid hormones is represented the body's remaining adaptive response to calories and protein by inducing hypothyroidism theoretically [60]. It was reported that the average level of rT3 was increased in patients with insulin-dependent diabetes, and the average metabolic clearance rate of rT3 is decreased [55, 61]. The result of Pittman CS et al. suggested that T4 monodeiodination of both phenyl rings was significantly impaired in uncontrolled diabetes, and they believed that long-term insulin insufficiency could lead to a more severe and extensive damage of T4 deiodination [55]. Type 2 deiodinase (Dio2) is an intracellular enzyme that catalyzes the conversion of T4 to T3 [62]. A meta-analysis showed that the polymorphism of Dio2 Thr92Ala is associated with poor blood glucose control in patients with T2DM [63].

The limitation of this study is that meta-analysis is a secondary literature analysis based on previous research evidence, so there are limitations and bias in the analysis. The study lacked data for long-term follow-up. The methods used to measure thyroid hormones were much less sensitive than those used in the last decade.

## 5. Conclusion

Thyroid function changed in patients with DKA. It changed with the severity of DKA. This condition may be transient, preceding further recovery of DKA.

## Figures and Tables

**Table 1:** Basic characteristics of included studies.

Author	Diagnosis of DKA	Determination of thyroxine	Inclusion criteria	Exclusion criteria	Therapeutic method	Treatmenttime	Subgroup
Yan Zhao	NA	https://fanyi.baidu.com/, https://fanyi.baidu.com/, https://fanyi.baidu.com/-zh/en/javascript:void(0), chemiluminescence	There was no thyroid disease in the past, and no drugs affecting thyroid function were taken recently	NA	Untreated		Mild: pH < 7.3 or HCO_3_^−^ < 15 mmol·L Moderate: pH < 7.2 or HCO_3_^−^ < 10 mmol·L Severe: pH < 7.1 or HCO_3_^−^ < 5 mmol·L
Shixiong Zhang		Microparticle automatic chemiluminescence immunoassay analyzer (Beckman, USA)	There was no abnormal ECG and liver function.	Patients with hypoproteinemia, thyroid disease, heart failure, fever, kidney disease, and acute viral hepatitis, as well as glucocorticoid, androgen, and estrogen were excluded	On the basis of routine diabetes treatment, the observation group was given routine treatment such as rehydration, removing inducement, maintaining acid-base and water-electrolyte balance, insulin, and other conventional treatment measures to correct ketoacidosis.	3 w	
Yunzhi Wang		Beckman microparticle automatic chemiluminescence immunoassay analyzer and corresponding kits provided by the company	The liver function and ECG were normal	Thyroid disease, acute viral hepatitis, hypoproteinemia, heart failure, kidney disease, infection, fever, pregnant women, and the use of estrogen, androgen, and glucocorticoid.	According to the treatment principle of ketoacidosis, the treatment includes removing the inducement, replenishing fluid, applying insulin, and maintaining the acid-base balance of water and electrolyte	3 w	
Yiping Wang		Beckman access 2 chemiluminescence immunoassay analyzer was used	There was no history of other acute and chronic diseases	Severe heart, liver, kidney, and connective tissue diseases Previous history of thyroid disease, taking thyroid function drugs Pregnant and lactating women	Treatment method unknown	unknown	Mild pH < 7.3 or HCO_3_^−^ < 15 mmol/L Moderate pH < 7.2 or HCO_3_^−^ < 10 mmol/L Severe pH < 7.1 or HCO_3_^−^ < 5 mmol/L
Lan Wang			Primary thyroid diseases, no history of antithyroid drugs and thyroid surgery were excluded		Treatment method unknown	unknown	
Yu Qiao	The symptoms of diabetes were aggravated, nausea, vomiting, dizziness, and other discomfort clinical manifestations Dry skin, sunken orbit, rapid pulse, and other signs Blood glucose >16 mmol/L, urine ketone body and urine sugar positive, blood gas analysis, anion gap increased, HCO_3_^−^decreased, and binding rate decreased (note: due to individual differences, some patients who have no obvious clinical symptoms or signs but meet the laboratory examination are also diagnosed with diabetic ketoacidosis)			Pregnant or lactating women Patients with thyroid disease history and taking drugs affecting thyroid function Patients with severe liver, heart, kidney, and connective tissue diseases; 40 patients with thyroid function analysis Patients with other crisis critical patients at admission Patients without the thyroid function test and blood gas analysis on admission.	Untreated		
Lianshan Piao		The immunoassay kit was provided by the Institute of Isotope, Chinese Academy of Atomic Energy	No pituitary, adrenal, and thyroid diseases were found, and no serious complications of chronic diabetes were found		After the treatment of high-dose rehydration and low-dose insulin continuous intravenous therapy	Urinary ketone body turned negative and carbonate ion returned to normal	
Li Luo		Siemens Centaur XP chemiluminescence immunoassay system		Other diseases that may affect thyroid function were excluded, and drugs affecting thyroid function were excluded	Untreated		
Shengbin Liu				Combined with serious heart, brain, liver, kidney, and other organ damage, thyroid disease, central nervous system systemic diseases, pregnant women having dopamine, glucocorticoid, androgen, and estrogen within 3 months may affect their own hormone levels, thus interfering with the drug use history of this study and having suffered from endocrine system diseases such as primary aldosteronism, and growth retardation	Untreated		Mild (pH ≥ 7.3) Moderate (7.3>pH ≥ 7.2) Severe (pH < 7.2)
Bin Liu			There was no history of thyroid disease, endocrine, glucocorticoid, sedative, furosemide, dopamine, and other drugs in the past, except lactation and pregnancy women.		Untreated		
Shaohui Huang		Microparticle automatic chemiluminescence analyzer (Beckman company, USA)	All the patients met the diagnostic criteria of diabetes established by the WHO	Abnormal ECG, abnormal liver function, the history of glucocorticoid, androgen, thyroid disease, infection, heart failure, and mental disease	Active treatment of primary disease, adequate fluid supplement, insulin, correction of water-electrolyte balance disorder, acid-base balance, and symptomatic treatment measures were adopted.	3 w	
Rui Feng	Blood glucose was higher than 13.9 mmol/L, pH was less than 7.35, urine ketone was positive, anion gap was more than 16 mmol/L, and blood bicarbonate (HCO_3_^−^) was less than 18 mmol/L	Enzyme linked immunosorbent assay		Ketoacidosis caused by acute cardiovascular and cerebrovascular diseases, gastrointestinal bleeding, major surgery, and pregnancy were excluded	All patients were treated with antibiotics to prevent infection, supplement electrolytes, and maintain body fluid balance. Patients with other basic diseases or complications were treated according to their condition. On this basis, the patients were treated with low-dose insulin intravenous drip, and the dose was 4–6 u/h	24 h	
Wen Fan		Abbott i2000 chemiluminescence immunoassay system and its kit	Age ≥65 years. According to the diagnostic criteria issued by the American Diabetes Association in 2010. Informed consent in this study.	There are hypothyroidism diseases, such as graves' disease and Hashimoto's thyroiditis. Those who have recently taken drugs that affect thyroid function, such as estrogen, androgen, and glucocorticoid. Combined with acute viral hepatitis, hypoproteinemia, heart failure, kidney disease, infection, and so on.	Treatment method unknown	Unknown	
Fuwan Ding		Radioimmunoassay		Patients with the history of thyroid disease, severe heart, liver, kidney disease, and connective tissue were excluded.	Resuscitation measures such as fluid rehydration, use of insulin to lower blood sugar, correction of water-electrolyte and acid-base imbalance, treatment of complications and comorbidities, and removal of ketosis inducements have stabilized the condition within 1–3 days. All patients did not use thyroxine preparations	2 w	
Qing Chen		Chemiluminescence		The history of thyroid diseases, patients taking drugs that affect thyroid function, severe heart, liver, kidney, and connective tissue diseases, breast-feeding, and pregnant women	untreated		
Daoxiong Chen	Diabetes (according to the WHO's diagnostic criteria for diabetes) Positive blood ketones Blood gas analysis showed metabolic acidosis		None of the observed patients had clinical manifestations of hyperthyroidism or hypothyroidism and no history of thyroid disease		Treatment method unknown	2 w	
Rashidi. H			Without any history of thyroid problems, systemic diseases, and using drugs which interfere with thyroid function were enrolled into the study.		Treatment method unknown	2w	
Naeije. R. G					Untreated	5 days	
Schienger. J. L			Clinically euthyroid		Untreated		
Alexander 1983		Double antibody RIA commercial method (Abbott Laboratories, North Chicago, IL).		We limited the scope of our study to the effects of diabetes mellitus per se by excluding patients with other systemic illnesses	Insulin	5 days	
Miboluk. AA		Two different methods: radio immune assay (RIA) and immune-radiometric assay (IRMA)	Blood sugar> 300 mg/dl, HCO_3_^−^ ≤ 15 mol/l PH ≤ 7.3, urine ketone positive	Severe nutritional deficiency, neurologic side effects, and brain edema/coma in ketoacidotic status	Insulin	5 days	
Lin. C. H	Serum glucose level 300 mg/dl (16.7 mmol/L), a serum pH < 7.25 or serum bicarbonate < 15 mmol/L, and the presence of ketones in the urine.	T3 was measured by radioimmunoassay (ICN, New York, USA; reference range, 100 to 190 ng/dl), T4 by radioimmunoassay (Daiichi, Tokyo, Japan; reference range, 4.4–12.5 *μ*g/dl), TSH by radioimmunometric assay (Daiichi, Tokyo, Japan; reference range, 0.5–5.15 IU/ml), and free T4 by radioimmunoassay, using the 125I-labeled T4 analogue method (DPC, Los Angeles, USA; reference range, 0.8–2.0 ng/dl).	Clinically euthyroid			3 days	
Jiao W	Blood glucose level >13.9 mmol/L, blood pH < 7.35, ketonuria positivity, anion gap (AG) > 16 mmol/L, and HCO_3_^−^ level < 18 mmol/L	Enzyme linked immunosorbent assay (ELISA)		Patients with DKA induced by acute cardiovascular and cerebrovascular diseases, gastrointestinal haemorrhage, major surgery, or pregnancy were excluded	Supportive treatment such as fluid infusion, acid-base imbalance correction, and electrolyte disturbance corintravenous insulin administered by an insulin pump at a rate of 4–6 U/h.	24 h	
Hu Y Y	Blood glucose (BG) > 11 mmol/L, venous pH < 7.3, or bicarbonate < 15 mmol/L	Automated chemiluminescent immunoassay system (Advia Centaur, Siemens, Munich, Germany).		Excluded patients with other endocrinological disorders, systemic illness, pituitary and thyroid disease, and a history of diabetes mellitus. Patients who had previously received any medication apart from insulin were also excluded	After resolution of DKA, patients received multiple daily insulin injections, aspart (Novo Nordisk, Bagsvaerd, Denmark) immediately before each meal and glargine (Sanofi-Aventis, Paris, France) once daily at bedtime. The total daily insulin dose ranged from 0.6 to 1.5 IU/kg.	7 days	
D. Glinoer, R		Serum FT 4 was measured using the kinetic FT4-I125 radioimmunoassay test system (kindly provided by Dr. G. Odstrchel, Corning Glass Works, Corning, NY, USA)			Low-dose insulin. Fluids and electrolytes	5 days	
F. Chiarelli	pH < 7.2, HCO_3_^−^ < 15 mmol/1, ketonuria: 4+).		Without familiar or personal history for endocrinological diseases. No drugs (except insulin for the diabetics) were administered to the children. All the subjects examined were clinically euthyroid, and their weight did not exceed ideal body by more than 20%.		Untreated		
Alexander, 1982		Double antibody RIA			Untreated		
Xin Y	Hyperglycaemia above 14 mmol/L and pH < 7.3 or bicarbonate < 15 mmol/L in the presence of ketonuria				Untreated		

**Table 2:** Comparison of thyroid function before and after treatment in patients with diabetes and DKA.

Author	Year	Country	DKA			After treatment		
			Mean	SD	*n*	Mean	SD	*n*
T4								
Daoxiong et al. [10]	1999	China	79.38	19.13	65	78.32	16.5	65
Glinoer et al. [11]	1980	Belgium	6.2	0.6	17	9.7	0.7	17
Wang [12]	1999	China	110.26	45.89	62	118	48.57	62
Piao and Li [13]	1999	China	71.4	12.6	8	70.9	12.3	8
Lin et al. [14]	2003	China	4.39	3.03	76	7.72	2.29	76
Mirboluk et al. [15]	2017	Iran	3.18	1.4	16	5.17	2.4	16
Naeije et al. [16]	1978	Germany	5.7	3.05	19	8.9	2.18	19
Rashidi et al. [17]	2017	Iran	7.6	2.53	20	8.41	2.51	20
Huang and Su [18]	2016	China	96.41	5.12	63	110.34	8.32	63
Fan [19]	2014	China	95.83	7.54	81	102.54	8.04	81
Wang and Du [20]	2013	China	96.3	23.1	69	109	37.9	69
Zhang [21]	2014	China	95.8	23.2	74	110.3	38.6	74
Ding and Ji [22]	2011	China	96.29	20.1	30	102.3	20.55	30
Wang et al. [23]	2018	China	65.68	20.32	40	90.33	20.95	40

T3								
Daoxiong et al. [10]	1999	China	1	0.24	65	1.16	0.21	65
Wang [12]	1999	China	1.26	0.46	62	2.29	0.59	62
Piao and Li [13]	1999	China	51.43	3.51	8	82.37	2.58	8
Lin et al. [14]	2003	China	59.36	36.11	76	140.63	48.24	76
Mirboluk et al. [15]	2017	Iran	63.2	28.2	16	78.5	26.2	16
Naeije et al. [16]	1978	Germany	37	6	19	105	9	19
Rashidi et al. [17]	2017	Iran	86	25.7	20	161.25	38	20
Huang and Su [18]	2016	China	1.34	0.25	63	1.65	0.31	63
Fan [19]	2014	China	1.38	0.12	81	1.45	0.21	81
Wang and Du [20]	2013	China	1.38	0.23	69	1.52	0.39	69
Zhang [21]	2014	China	1.39	0.24	74	1.53	0.41	74
Ding and Ji [22]	2011	China	0.75	0.2	30	1.68	0.33	30
Wang et al. [23]	2018	China	0.84	0.3	40	1.55	0.33	40

FT4								
Daoxiong et al. [10]	1999	China	14.21	2.8	65	13.8	2.5	65
Glinoer et al. [11]	1980	Belgium	1.4	0.1	17	1.7	0.1	17
Hu et al. [24]	2015	China	11.38	3.58	40	15.57	2.92	40
Jiao et al. [25]	2016	China	11.61	3.53	120	14.23	3.01	120
Lin et al. [14]	2003	China	0.59	0.36	76	1.29	0.32	76
Rashidi et al. [17]	2017	Iran	1.07	0.43	20	1.58	0.62	20
Feng [26]	2014	China	11.62	3.52	60	14.24	3.03	60
Huang and Su [18]	2016	China	13.21	0.24	63	15.28	0.65	63
Fan [19]	2014	China	13.44	0.95	81	14.02	1.21	81
Wang and Du [20]	2013	China	13	2.3	69	14.2	1	69
Zhang [21]	2014	China	13.2	2.4	74	14.6	1.1	74
Ding and Ji [22]	2011	China	13.32	2.52	30	13.92	3.99	30
Wang et al. [23]	2018	China	11.91	2.85	40	14.26	2.47	40

FT3								
Daoxiong et al. [10]	1999	China	2.48	0.9	65	3.38	0.98	65
Hu et al. [24]	2015	China	2.63	0.58	40	4.77	1.15	40
Jiao et al. [25]	2016	China	2.85	1.22	120	3.98	1.02	120
Rashidi et al. [17]	2017	Iran	147	0.4	20	3.8	0.86	20
Feng [26]	2014	China	2.87	1.23	60	3.96	1.03	60
Fan [19]	2014	China	3.54	0.23	81	3.6	0.34	81
Wang and Du [20]	2013	China	3.54	0.53	69	3.69	0.51	69
Zhang [21]	2014	China	3.55	0.54	74	3.65	0.48	74
Ding and Ji [22]	2011	China	2.21	0.41	30	4.08	0.55	30
Wang et al. [23]	2018	China	2.71	0.83	40	4.48	0.67	40

TSH								
Daoxiong et al. [10]	1999	China	1.95	0.85	65	1.74	0.87	65
Glinoer et al. [11]	1980	Belgium	29	61	17	74	82	17
Hu et al. [24]	2015	China	1.77	1.19	40	2.17	0.91	40
Jiao et al. [25]	2016	China	1.8	0.76	120	2.33	0.87	120
Wang [12]	1999	China	2.94	2.07	62	3.21	2.35	62
Lin et al. [14]	2003	China	1.37	1.46	76	2.03	1.29	76
Mirboluk et al. [15]	2017	Iran	1.85	1.5	16	1.79	1.3	16
Naeije et al. [16]	1978	Germany	1.9	1.3	19	2.6	1.3	19
Feng [26]	2014	China	1.82	0.75	60	2.32	0.86	60
Huang and Su [18]	2016	China	4.48	1.24	63	4.09	1.03	63
Fan [19]	2014	China	4.08	0.28	81	3.89	0.43	81
Wang and Du [20]	2013	China	4.47	1.59	69	4.12	1.47	69
Zhang [21]	2014	China	4.49	1.61	74	4.24	1.51	74
Ding and Ji [22]	2011	China	1.33	0.76	30	1.56	0.77	30
Wang et al. [23]	2018	China	1.75	1.28	40	2.63	1.18	40

T3RU								
Glinoer et al. [11]	1980	Belgium	31.1	4.5	17	29.4	5.8	17
Lin et al. [14]	2003	China	33.33	4.52	76	32.07	4.31	76
Mirboluk et al. [15]	2017	Iran	32.4	1.8	16	32.1	1.5	16
Naeije et al. [16]	1978	Germany	31.5	4.4	19	30.8	6.5	19
Rashidi et al. [17]	2017	Iran	1.7	0.46	20	4.05	0.77	20

rT3								
Ding and Ji [22]	2011	China	1.3	0.3	30	0.81	0.16	30
Naeije et al. [16]	1978	Germany	40	26.15	19	24	26.15	19
Daoxiong et al. [10]	1999	China	0.78	0.09	65	0.49	0.09	65

**Table 3:** Comparison of thyroid function between patients with diabetes with and without DKA.

Author	Year	Country	DKA			Control		
			Mean	SD	*n*	Mean	SD	*n*
T4								
Alexander et al. [27]	1983	United States	5.5	0.6	12	8.7	0.6	6
Chiarelli et al.[28]	1989	Germany	58.22	15.02	16	74.04	23.07	45
Daoxiong et al. [10]	1999	China	79.38	19.13	65	94.6	18.12	60
Li et al. [29]	2012	China	82.4289	22.6743	38	109.094	17.9297	36
Lin et al. [14]	2003	China	4.39	3.03	76	7.6	1.86	62
Schienger et al. [30]	1982	Germany	7.6	0.3	8	7.8	0.5	8
Huang and Su [18]	2016	China	96.41	5.12	63	107.52	8.12	62
Fan [19]	2014	China	95.83	7.54	81	106.45	9.09	94
Zhao et al. [31]	2012	China	5.65	2.8	91	9.28	2.85	110
Wang and Du [20]	2013	China	96.3	23.1	69	114.8	41.2	74
Qiao [32]	2012	China	5.95	1.57	40	7.8	1.67	40
S. Liu [33]	2016	China	92.9	18.78	23	114.09	18.83	31
Chen et al. [34]	2016	China	102.46	22.73	65	107.28	23.28	65
Zhang [21]	2014	China	95.8	23.2	74	165	42.3	84
Ding and Ji [22]	2011	China	96.29	20.1	30	100.3	20.33	30

T3								
Alexander et al. [27]	1983	United States	49.9	7.3	12	88	8	6
Alexander et al. [6]	1982	United States	49	9	4	103	7	11
Chiarelli et al.[28]	1989	Germany	1.04	0.36	16	1.39	0.42	45
Daoxiong et al. [10]	1999	China	1	0.24	65	1.25	0.36	60
Li et al. [29]	2012	China	1.5482	0.4371	38	1.9497	0.2762	36
Lin et al. [14]	2003		59.36	36.11	76	91.4	31.85	62
Schienger et al. [30]	1982	Germany	124	14	8	115	6	8
Huang and Su [18]	2016	China	1.34	0.25	63	1.52	0.31	62
Fan [19]	2014	China	1.38	0.12	81	1.55	0.21	94
Zhao et al. [31]	2012	China	0.54	0.51	91	1.02	0.38	110
Wang and Du [20]	2013	China	1.38	0.23	69	1.59	0.47	74
Qiao [32]	2012	China	0.75	0.22	40	1.05	0.21	40
S. Liu [33]	2016	China	1.6	0.41	23	1.85	0.33	31
Chen et al. [34]	2016	China	1.53	0.24	65	1.72	0.27	65
Zhang [21]	2014	China	1.39	0.24	74	1.61	0.45	84
Ding and Ji [22]	2011		0.75	0.2	30	1.72	0.31	30

FT4								
Liu [35]	2012	China	0.84	0.21	20	1.15	0.38	60
Chiarelli et al.[28]	1989	Germany	10.24	2.94	16	11.55	3.62	45
Daoxiong et al. [10]	1999	China	14.21	2.8	65	12.13	2.88	60
Qiu et al. [36]	2018	China	12.4	4.89	75	15.97	3.08	39
Lin et al. [14]	2003	China	0.59	0.36	76	1.18	0.4	62
Huang and Su [18]	2016	China	13.21	0.24	63	14.29	0.31	62
Fan [19]	2014	China	13.44	0.95	81	14.35	0.36	94
Xin et al. [37]	2010	China	12.99	7.3	85	15.34	3.97	118
Wang and Du [20]	2013	China	13	2.3	69	14	1.2	74
Qiao [32]	2012	China	1.13	0.26	40	1.21	0.17	40
S. Liu [33]	2016	China	10.76	2.1	23	12.12	1.46	31
Chen et al. [34]	2016	China	11.86	2.57	65	12.51	2.78	65
Zhang [21]	2014	China	13.2	2.4	74	14.9	1.3	84
Ding and Ji [22]	2011	China	13.32	2.52	30	14.31	4.01	30
Schienger et al. [30]	1982	Germany	2.3	0.29	8	2.2	0.29	8

FT3								
Liu [35]	2012	China	3.96	0.92	20	5.83	1.96	60
Chiarelli et al.[28]	1989	Germany	2.05	1.01	16	2.35	0.71	45
Daoxiong et al. [10]	1999	China	2.48	0.9	65	3.46	0.89	60
Qiu et al. [36]	2018	China	2.47	0.74	75	3.07	0.91	39
Fan [19]	2014	China	3.54	0.23	81	3.69	0.44	94
Xin et al. [37]	2010	China	2.61	1.93	85	3.31	1.27	118
Wang and Du [20]	2013	China	3.54	0.53	69	3.65	0.49	74
Qiao [32]	2012	China	2.21	0.61	40	2.85	0.3	40
S. Liu [33]	2016	China	4.32	0.66	23	4.95	0.63	31
Chen et al. [34]	2016	China	3.95	1.14	65	4.46	1.17	65
Zhang [21]	2014	China	3.55	0.54	74	3.64	0.51	84
Ding and Ji [22]	2011	China	2.21	0.41	30	4.25	0.41	30

TSH								
Alexander et al. [27]	1983	The United States	3.4	0.9	12	4.4	0.7	6
Liu [35]	2012	China	0.83	0.73	20	1.25	1.19	60
Chiarelli et al.[28]	1989	Germany	1.66	0.69	16	2.56	1.27	45
Daoxiong et al. [10]	1999	China	1.95	0.85	65	2.02	0.96	60
Li et al. [29]	2012	China	1.5092	1.3515	38	2.0213	0.9604	36
Lin et al. [14]	2003	China	1.37	1.46	76	1.6	1.01	62
Huang and Su [18]	2016	China	4.48	1.24	63	4.46	1.31	62
Fan [19]	2014	China	4.08	0.28	81	3.95	0.23	94
Xin et al. [37]	2010	China	1.7	1.48	85	1.66	0.77	118
Zhao et al. [31]	2012	China	2.49	2.73	91	2.45	2.01	110
Wang and Du [20]	2013	China	4.47	1.59	69	4.23	1.53	74
Qiao [32]	2012	China	1.38	0.86	40	1.82	0.88	40
S. Liu [33]	2016	China	1.27	1.04	23	2.17	1.33	31
Chen et al. [34]	2016	China	3.15	0.58	65	3.17	0.71	65
Zhang [21]	2014	China	4.49	1.61	74	4.23	1.5	84
Ding and Ji [22]	2011	China	1.33	0.76	30	1.43	0.82	30

rT3								
Ding and Ji [22]	2011	China	1.3	0.3	30	0.83	0.17	30
Chiarelli et al.[28]	1989	Germany	0.23	0.1	16	0.22	0.07	45
Alexander et al. [27]	1983	The United States	57.8	25.3	12	35.7	8.3	6
Schienger.J.B	1982	Germany	25.2	3.4	8	23.5	5.4	8
Alexander et al. [6]	1982	The United States	83	2	4	33	2	35
Daoxiong et al. [10]	1999	China	0.78	0.09	65	0.52	0.1	70

## Data Availability

The data used to support the findings of this study are included within the article.
