# Corrigendum to “Impact of Diabetic Ketoacidosis on Thyroid Function in Patients with Diabetes Mellitus”

**DOI:** 10.1155/2021/9832382

**Published:** 2021-05-26

**Authors:** Yuling Xing, Jinhu Chen, Guangyao Song, Liying Zhao, Huijuan Ma

**Affiliations:** ^1^Department of Endocrinology, Hebei General Hospital, Shijiazhuang 050017, China; ^2^Graduate School of Hebei Medical University, Shijiazhuang 050017, China; ^3^Hebei Key Laboratory of Metabolic Diseases, Hebei General Hospital, Shijiazhuang 050051, Hebei, China; ^4^Department of Internal Medicine, Hebei Medical University, Shijiazhuang 050017, Hebei, China

In the article titled “Impact of Diabetic Ketoacidosis on Thyroid Function in Patients with Diabetes Mellitus” [1], the authors identified an error in the presentation of the flowchart in Figure 1, and the corrected figure is as follows:

The authors and the editorial board agree to the publication of the corrigendum.

## Figures and Tables

**Figure 1:**
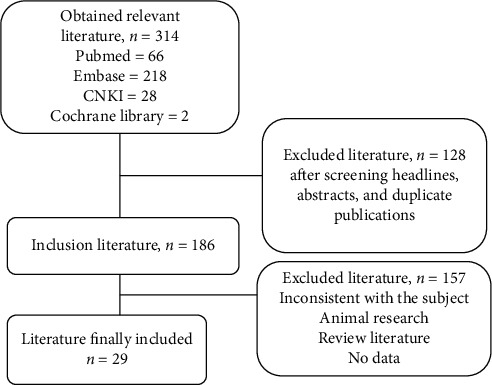
The process of study selection.
